# Short-Term Effect of Laser Acupuncture on Lower Back Pain: A Randomized, Placebo-Controlled, Double-Blind Trial

**DOI:** 10.1155/2015/808425

**Published:** 2015-10-01

**Authors:** Jae-Young Shin, Boncho Ku, Jaeuk U. Kim, Yu Jung Lee, Jae Hui Kang, Hyun Heo, Hyo-Joon Choi, Jun-Hwan Lee

**Affiliations:** ^1^Clinical Research Division, Korea Institute of Oriental Medicine, Daejeon 34054, Republic of Korea; ^2^KM Fundamental Research Division, Korea Institute of Oriental Medicine, Daejeon 34054, Republic of Korea; ^3^Technology Licensing & Commercialization Team, Korea Institute of Oriental Medicine, Daejeon 34054, Republic of Korea; ^4^Department of Acupuncture & Moxibustion Medicine, College of Oriental Medicine, Daejeon University, Daejeon 34520, Republic of Korea; ^5^Solco Biomedical Co., Ltd., Pyeongtaek, Gyeonggi 17704, Republic of Korea

## Abstract

*Purpose*. This trial was performed to investigate the efficacy of laser acupuncture for the alleviation of lower back pain. * Methods*. This was a randomized, placebo-controlled, double-blind trial. Fifty-six participants were randomly assigned to either the laser acupuncture group (*n* = 28) or the sham laser acupuncture group (*n* = 28). Participants in both groups received three treatment sessions over the course of one week. Thirteen acupuncture points were selected. The visual analogue scale for pain, pressure pain threshold, Patient Global Impression of Change, and Euro-Quality-of-Life Five Dimensions questionnaire (Korean version) were used to evaluate the effect of laser acupuncture treatment on lower back pain. *Results*. There were no significant differences in any outcome between the two groups, although the participants in both groups showed a significant improvement in each assessed parameter relative to the baseline values. *Conclusion*. Although there was no significant difference in outcomes between the two groups, the results suggest that laser acupuncture can provide effective pain alleviation and can be considered an option for relief from lower back pain. Further studies using long-term intervention, a larger sample size, and rigorous methodology are required to clarify the effect of laser acupuncture on lower back pain.

## 1. Introduction

Lower back pain (LBP) is one of the most common pathological conditions, affecting 80–85% of individuals at least once during their lifetime and exhibiting a yearly prevalence rate of approximately 15–45%. Aggravation of this pain, which is a frequent occurrence, leads to serious social and economic burdens for patients and limits their activity [1].

The “obstruction of qi and blood in the meridian” is one of the major causes of LBP [2]. Acupuncture stimulates specific points to relieve obstruction in the channels, rectify qi, and stimulate blood circulation [3]. Accordingly, it is commonly used to alleviate LBP and is an effective treatment for this condition. However, patients may be reluctant to accept this treatment because it requires frequent visits to the doctor. In addition, patients fear the possibility of pain and inflammation related to the use of needles. For pain relief, patients may instead use ointments or plasters, which are convenient but do not provide any obvious or long-lasting effects.

Therefore, it is necessary to develop new equipment as an effective replacement for conventional acupuncture equipment. The technology used to stimulate acupuncture points has advanced, shifting from stone to metal, and has now advanced to laser acupuncture [4]. The use of laser light as an alternative to stimulate acupuncture points has been promoted for almost three decades [5]. Generally referred to as laser acupuncture, it has been clinically applied since the 1970s and was made possible by the application of a low-intensity laser [4]. Previous studies have shown that laser acupuncture can stimulate acupuncture points for neck pain treatment [6]. Furthermore, low level laser therapy (LLLT) on wound areas as well as on acupuncture points, as a noninvasive, pain-free method with minor side effects, has been considered as a possible treatment option for the diabetic foot syndrome [7]. In addition, several studies have reported that laser acupuncture is effective for the treatment of many diseases, such as chronic tension headache, fibromyalgia, and chronic neck pain [8–10].

The aim of this study was to develop a convenient and safe laser acupuncture technique with satisfactory efficacy for the treatment of lower back pain and to record its therapeutic effects.

## 2. Methods

### 2.1. Study Design

We conducted a randomized, placebo-controlled, double-blind parallel study to compare the effect of sham laser acupuncture with laser acupuncture. The study was carried out at the Department of Acupuncture & Moxibustion, Cheonan Korean Medicine Hospital of Daejeon University, Republic of Korea.

**Figure 1:**
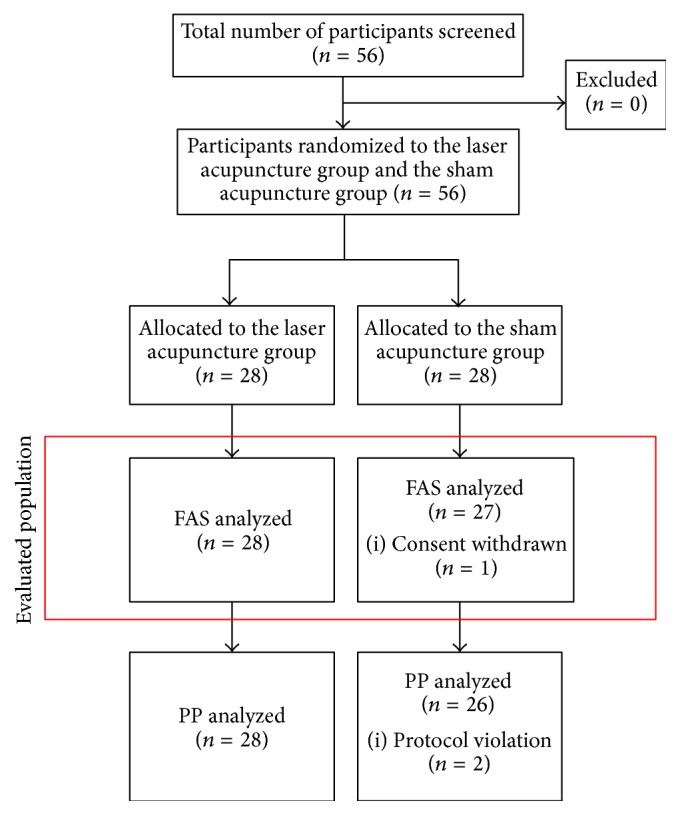
Study flowchart of the laser acupuncture randomized, placebo-controlled, double-blind trial for lower back pain. FAS: full analysis set; PP: per protocol.

Fifty-six participants with LBP were recruited through advertisement. Following radiographic diagnosis, each participant received an explanation of the study procedure and signed the consent form. Vital signs and relevant histories were recorded. The participants were randomly assigned to either the laser acupuncture group (*n* = 28) or the sham laser acupuncture group (*n* = 28), and they received laser or sham laser stimuli at five bilateral acupuncture points and three midline acupuncture points for three minutes. Every participant received three treatment sessions over the course of one week. The treatment interval ranged from two to four days. Interviews and clinical examinations were conducted within one week after the last treatment session. The study design is depicted in Figure 1.

### 2.2. Randomized Allocation and Blinding

The participant randomization list was compiled by an external investigator and was not divulged to other investigators or the participants. The external investigator prepared a series of sealed, sequentially numbered envelopes containing the treatment assignments. When a participant fulfilled the inclusion criteria, the study investigator opened the lowest numbered envelope to reveal the group allocation. Blinding was achieved for both the investigator and the participant, without revealing either the laser acupuncture or the placebo acupuncture treatment.

### 2.3. Participants

The inclusion criteria for the men and women in the study were as follows: (1) age range of 20 to 75 years; (2) minimum pain intensity of 30 mm on the visual analogue scale (VAS) for pain, which ranges from 0 to 100 mm; (3) a diagnosis of lower back pain; (4) the ability to participate voluntarily, clearly understand the purpose and characteristics of the clinical trial, and sign an informed consent form. Participants were excluded from the study if one or more of the following criteria were fulfilled: (1) the use of devices such as pacemakers and hearing aids that could be affected by electromagnetic fields; (2) presence of severe pain that precluded participation in a clinical trial; (3) diagnoses of fractures, severe disc herniation, or spinal tumors, which require immediate examination and treatment; (4) use of medication, such as corticosteroids, anticonvulsants, and anti-inflammatory drugs that could affect the outcome of the trial; (5) pregnancy; (6) prior history of adverse effects, to physical stimulation therapy; (7) significant physical or mental deficiencies preventing a clear understanding of the clinical trial procedure; (8) participation in other clinical trials within the previous month.

### 2.4. Ethics Statement

The study protocol was approved by the Ethics Committee of Cheonan Korean Medicine Hospital of Daejeon University, Korea. Written informed consent was obtained from all participants in accordance with the Declaration of Helsinki.

### 2.5. Interventions

All interventions were conducted at the Department of Acupuncture & Moxibustion, Cheonan Korean Medicine Hospital of Daejeon University.

**Figure 2:**
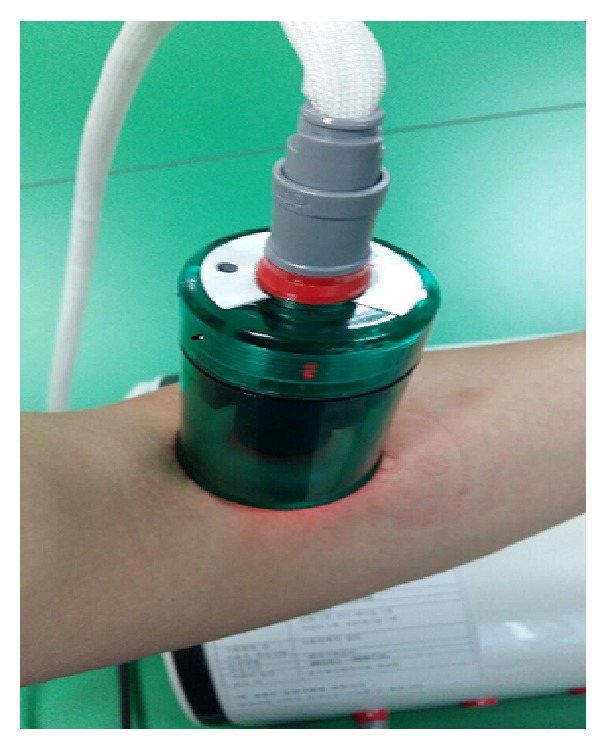
The intervention was performed with a cup-shaped laser acupuncture device.

**Figure 3:**
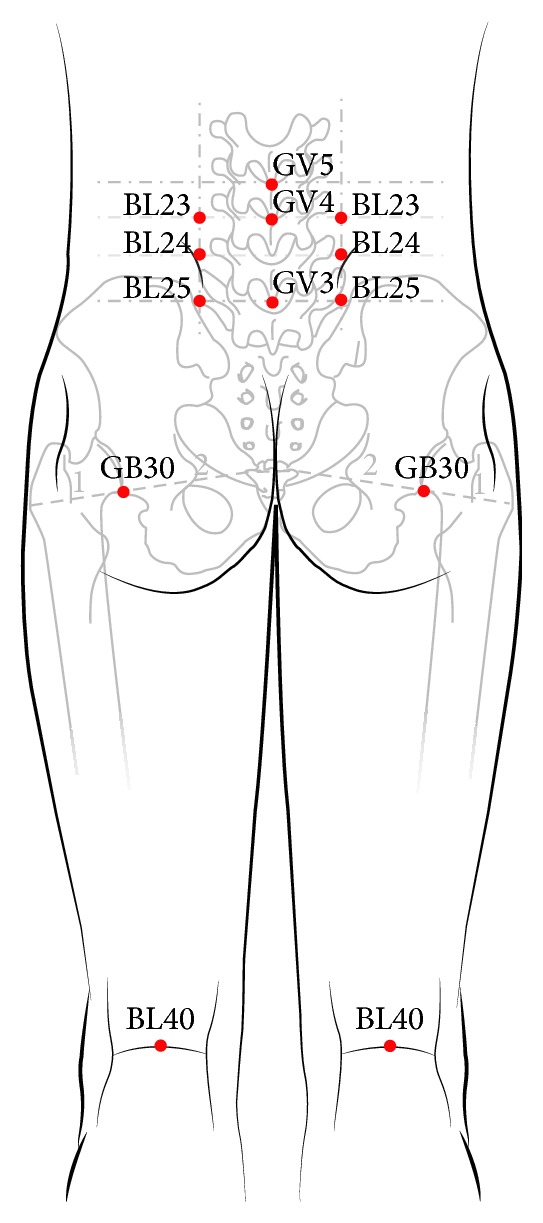
GV3, GV4, and GV5 and both sides of BL23, BL24, BL25, BL40, and GB30 (13 acupuncture points).

Laser acupuncture (a combination of medical laser irradiation equipment and cupping) was performed with an infrared 4-channel laser of low intensity (Solco-LF100, Solco Biomedical Co., Ltd., Pyeongtaek, Korea) (Figure 2). The maximum output power was 53 mW with a laser light wavelength of 660 nm (pulse-type wave). The irradiated area of the skin was 2 × 2 mm^2^, frequency was 200 Hz, duty ratio was 50%, pressure was 15 kPa, and output power was 50 mW. Each treatment session lasted three minutes. This laser device is also equipped by the manufacturer with visual (red, light-emitting diode) and acoustic signals. Treatment was performed in sequence at 13 commonly used acupuncture points: unilateral GV3, GV4, and GV5 and bilateral BL23, BL24, BL25, BL40, and GB30 (Figure 3). The distance between the skin and laser was 5 mm. The acupuncture points were localized without tactile irritation.

The sham laser acupuncture group underwent the same procedure as the laser acupuncture group, but the laser was not turned on. The acoustic functions of the equipment were preserved to ensure blinding. The sham laser acupuncture was performed according to the medical laser irradiation equipment treatment protocol.

We recorded the visual analogue scale (VAS) for pain and the pressure pain threshold (PPT) during visits 1–4 and measured the patient global impression of change (PGIC) and the Korean version of Euro-Quality-of-Life Five Dimensions (EQ-5D) at visits 1 and 4.

During the clinical trial period, other treatments for spinal sprain (except as prescribed in the protocol) were prohibited. The study institution offered a kinesiology guidebook to all participants to use for daily exercise therapy for LBP.

### 2.6. Outcome Measures

#### 2.6.1. Visual Analogue Scale (VAS) for Pain

The 100 mm VAS is an instrument for the self-assessment of pain. Each participant marked the level of pain, and the VAS score was determined by measuring the distance between the left-hand end of the line and the marked point. We recorded VAS scores during visits 1–4. The scores reported at visit 1 were used for screening purposes and as the baseline measurements.

#### 2.6.2. Pressure Pain Threshold (PPT)

The PPT is defined as the minimum applied force that induces pain. The investigator placed a pressure algometer on BL25, instructed participants to indicate the first sensation of slight pain either vocally or by raising their hands, and then measured the pressure (kg/cm^2^). We recorded PPT scores during visits 1–4. The scores obtained at visit 1 were used as the baseline measurements.

#### 2.6.3. Patient Global Impression of Change (PGIC)

The PGIC evaluates the changes (if any) in activity limitations, symptoms, emotions, and overall quality of life which are related to painful conditions, as described by the participants from the beginning of the treatment. Each dimension has seven levels: (1) no change, or the condition is worse; (2) almost the same, with little change; (3) better, but no noticeable change; (4) somewhat better, but the change has made no real difference; (5) moderately better, with a slight but noticeable change; (6) better, with a definite improvement that has made a real and worthwhile difference; (7) a great deal better, with a considerable improvement that has made a marked difference. Participants chose the level that matched the degree of change. We assessed the PGIC at visits 1 and 4.

#### 2.6.4. Euro-Quality-of-Life Five Dimensions (EQ-5D, Korean Version)

The EQ-5D is a standardized measure of health status developed by the EuroQol Group in order to provide a simple, generic measure of health for clinical and economic appraisal. The EQ-5D consists of two parts: the EQ-5D descriptive system and the EQ visual analogue scale (EQ VAS). The EQ-5D descriptive system comprises the following five dimensions: mobility, self-care, usual activities, pain/discomfort, and anxiety/depression. Each dimension has three levels: no problems, some problems, and severe problems. Respondents were asked to indicate their health state by ticking (or placing a cross) in the box against the most appropriate statement in each of the five dimensions. We gave participants this questionnaire at visits 1 and 4.

### 2.7. Statistical Analysis

The sample size was calculated using the conventional power analysis method. Twenty- eight participants were recruited in each group.

The software package R (version 3.0.1, “R & R” of the Statistics Department of the University of Auckland, Auckland, New Zealand) was used for statistical analyses. Efficacy measurements were adjusted by the full analysis set. Missing values were input by using the last observation carried forward method.

The intergroup comparison of means (before and after the intervention) was processed by the analysis of covariance (ANCOVA), and the intragroup comparison of mean values (before and after the intervention) was analyzed by the paired *t*-test or the Wilcoxon signed rank sum test. The entire statistical analysis was a one-sided test. *P* < 0.05 was considered statistically significant.

### 2.8. Adverse Events

The investigator recorded adverse events and unexpected responses to laser acupuncture treatment. Adverse events were reported by the participants and evaluated by the investigator as mild, moderate, or severe according to the World Health Organization Draft Guidelines for Adverse Event Reporting [11] and Spilker's criteria [12].

## 3. Results

### 3.1. Demographic Characteristics and Baseline Values

In total, 56 participants with LBP were recruited for this study. All 28 participants assigned to the laser acupuncture group completed the study protocol. Of the 28 participants assigned to the sham laser acupuncture group, two failed to complete the protocol, as shown in Figure 1.

Participants in the two groups did not differ significantly in age, sex, duration, kinesitherapy, body mass index, diastolic blood pressure, pulse, or body temperature. However, systolic blood pressure differed significantly between participants in the groups (Table 1).

After collecting the data, we analyzed the results of the two participant groups. Baseline values (for VAS, PPT, PGIC, and EQ-5D) did not differ significantly between the two groups, as listed in Table 1.

### 3.2. Primary Outcome: Visual Analogue Scale (VAS) for Pain

**Figure 4:**
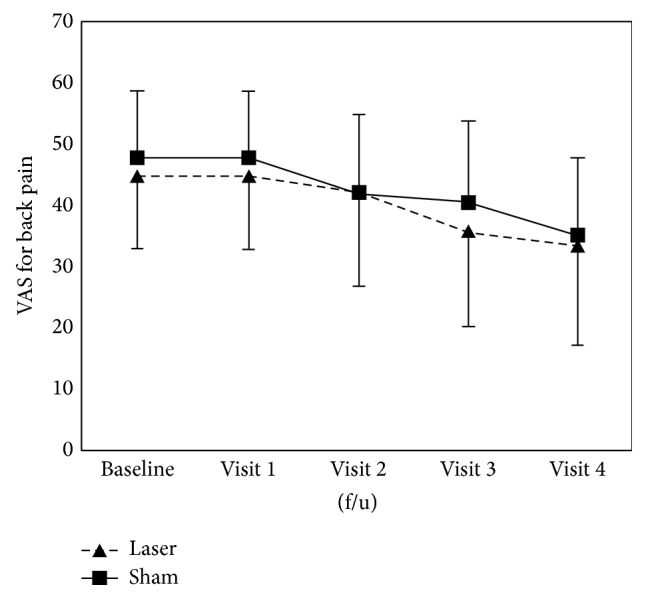
Change in visual analogue scale (VAS) for pain scores after intervention in both groups.

The VAS scores changed significantly between visit 1 (baseline) and visit 4 (*P* < 0.001) and also between visit 1 (baseline) and visit 4 (*P* < 0.001) in both groups. However, the intergroup comparison of mean values (before and after the intervention) did not reveal a significant difference in the VAS scores of the two groups (Figure 4 and Table 2).

### 3.3. Secondary Outcomes

**Figure 5:**
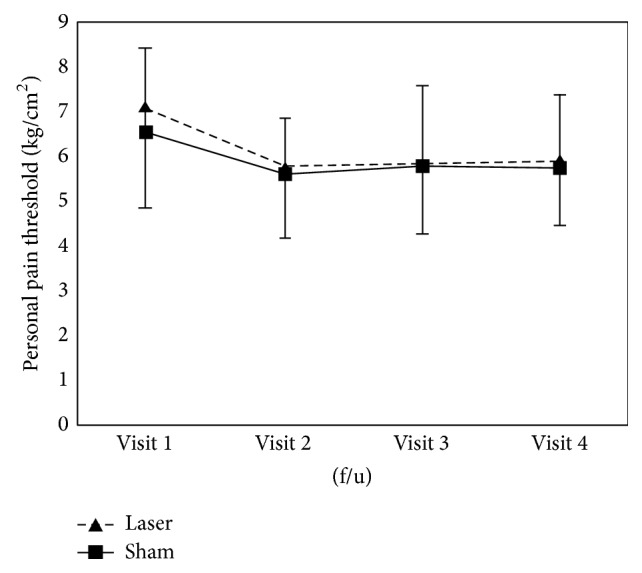
Change in pressure pain threshold (PPT) scores after intervention in both groups.

**Figure 6 fig6:**
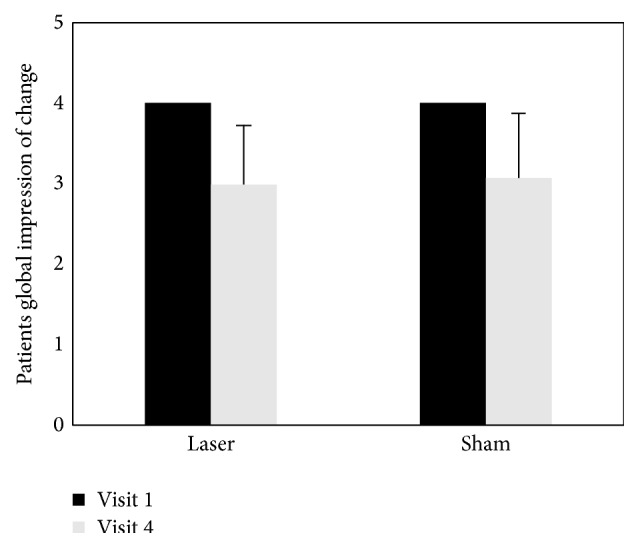
Change in patient global impression of change (PGIC) scores after intervention in both groups.

**Figure 7:**
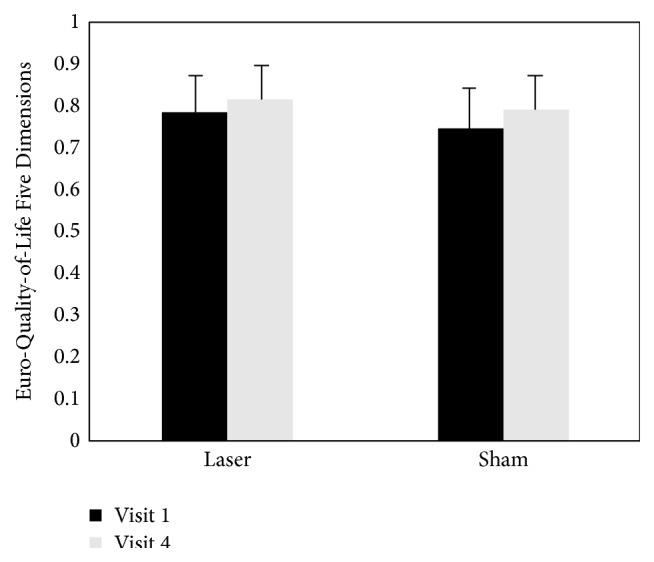
Change in Euro-Quality-of-Life Five Dimensions (EQ-5D, Korean Version) scores after intervention in both groups.

Data from the pain questionnaires are presented in Table 2. The results of secondary outcome measures were similar to those of the primary outcome measure. The PPT scores assessed at each time point compared with visit 1 (baseline) were significantly reduced in both groups (*P* < 0.01). The changes in PGIC between visit 1 (baseline) and visit 4 differed within both groups (*P* < 0.001). The EQ-5D questionnaire outcomes of both groups improved throughout the trial (laser acupuncture group, *P* < 0.05; sham laser acupuncture group, *P* < 0.01). The results reveal that the PPT, PGIC, and EQ-5D scores changed in both groups. However, we found no significant differences between the two groups in the secondary outcome measures (Figures 5 [Fig fig6]–7, Table 2).

### 3.4. Adverse Events

There were no indications of adverse effects (AE), such as erythema, itching, or blistering. No serious adverse events (SAE) were reported.

## 4. Discussion

### 4.1. Summary of Main Findings

The purpose of this study was to compare the efficacy of laser acupuncture with that of sham laser acupuncture for the treatment of LBP. Fifty-six participants with LBP were recruited and randomly assigned to either the laser acupuncture group or the sham laser acupuncture group. Participants in the two groups showed no significant differences in demographics; the significant difference in systolic blood pressure between the groups could have been incidental.

The intervention in the treatment group was performed with a cup-shaped laser acupuncture device (Figure 2); the same device with the laser turned off was used in control group. To treat LBP, acupuncture expert predefined a group of acupuncture points on 3 types of meridian (gallbladder meridian, GB; bladder meridian, BL; Governor Vessel, GV) according to the pain location. The Oriental Medicine is based on the theory that “There is stoppage, there is pain,” which means that pain is caused by the obstruction of qi and blood in the meridian. The traditional acupuncture-moxibustion theory states the following: “Rescue the waist and back from pain by the Weizhong (BL40).” On the basis of these theories, unilateral GV3, GV4, and GV5 and bilateral BL23, BL24, BL25, BL40, and GB30 are often used for pain relief of LBP (Figure 3). In this study, we recorded the VAS, PPT, PGIC, and EQ-5D of all participants before and after intervention. Although the scores improved in both groups, the improvement in symptoms was not significantly different between the laser acupuncture group and the sham laser acupuncture group. It is possible that suction of the cupping device stimulated the lesion locations. Cupping therapy by partial vacuum has been found to improve the local blood and lymphatic circulation, increase the temperature of the local skin, and relieve painful muscle tension [13]. To hold the device to the skin without causing physiologic or therapeutic effects, we designed the suction pressure within the cupping glass (15 kPa = 112.509 mmHg) to be weaker than that of normal cupping therapy (600 mmHg) [14]. However, we cannot exclude the possibility that cupping suction could trigger an analgesic effect. The development of a study control that is convenient and can also minimize physiologic effects will be important for future randomized controlled clinical trials for laser acupuncture.

### 4.2. Study Limitations

The participants in our study received only three treatments, because we sought to observe the short-term effects of laser acupuncture. According to one reference, approximately 10 treatment sessions are appropriate for acupuncture treatment [15]. In a systematic review of 16 laser acupuncture trials, the average number of treatment sessions was 9.6 [16]. Future studies on this topic are necessary to evaluate the optimum number of treatments.

The deqi, which is associated with the acupuncture effect, is one of the differences between acupuncture and placebo acupuncture [17]. Beissner and Marzolff reported that more than 80% of the participants experienced deqi after treatment by laser acupuncture [18]. Therefore, we suggest that further studies investigate the presence of deqi using questionnaire surveys to evaluate whether laser acupuncture could serve as an alternative to metal acupuncture and whether sham laser acupuncture is suitable for the control group.

Acupuncture textbooks suggest that the optimal depth of acupuncture stimulation in clinical treatment is 10.3–90.3 mm [15]. However, the laser wavelength in this study was fixed at 600 nm and penetrated approximately 5.5 mm into the acupuncture points, and it was impossible to change the depth of laser acupuncture stimulation. Because metal acupuncture needles are available in a variety of lengths and gauges, development of laser acupuncture devices with an adjustable wavelength and intensity of light should be considered.

## 5. Conclusion

This study compares laser acupuncture with sham laser acupuncture for the treatment of LBP. Substantial adverse events did not occur in the intervention period. Our results show that laser acupuncture is a relatively safe treatment that helps improve pain and the quality of life of patients with LBP. We recommend that future studies use long-term intervention, larger participant numbers, and rigorous methodology to determine the effect of laser acupuncture on LBP.

## Figures and Tables

**Table 1:** Baseline characteristics.

Characteristics	Group		*P* value
	The laser acupuncture group	The sham laser acupuncture group	
	(*n* = 28)	(*n* = 27)	
Age^†^	46.32 (10.2) [21, 47.5, 65]	46.48 (11.68) [23, 47, 68]	0.957
Sex^*∗*^			
Female	24 *〈*85.7*〉*	22 *〈*81.5*〉*	0.7287
Male	4 *〈*14.3*〉*	5 *〈*18.5*〉*	
Duration of back pain (month)^††^	13.78 (19.58) [0.2, 4.2, 75.19]	16.33 (28.13) [0.2, 6.18, 130.98]	0.8531
Compliance rate (%)^††^	100 (0) [100, 100, 100]	97.53 (12.83) [33.3, 100, 100]	0.3261
Kinesitherapy^††^	2.75 (0.44) [2, 3, 3]	2.56 (0.75) [0, 3, 3]	0.4192
BMI (kg/m^2^)^†^	26.05 (7.26) [16.4, 25.2, 59.19]	23.39 (3.05) [17.9, 23.26, 33.69]	0.0844
Systolic blood pressure (mmHg)^†^	122.96 (10.71) [97, 124.5, 143]	115.15 (11.48) [98, 114, 142]	0.0117
Diastolic blood pressure (mmHg)^†^	74.75 (10.55) [55, 76, 102]	70.3 (7.25) [59, 71, 89]	0.0748
Pulse (bpm)^†^	73.71 (10.3) [56, 71.5, 95]	74.96 (8.46) [61, 74, 90]	0.6259
Body temperature (°C)^†^	36.57 (0.36) [35.8, 36.5, 37.3]	36.47 (0.44) [35.4, 36.5, 37.4]	0.3398
Baseline value			
VAS^†^	44.64 (11.86) [30, 40, 75]	47.78 (10.95) [30, 40, 80]	0.3135
PGIC	4 (0) [4, 4, 4]	4 (0) [4, 4, 4]	
PPT^†^	7.12 (2.27) [3.1, 7.2, 11.92]	6.56 (1.86) [3.7, 6.23, 12.15]	0.3231
EQ-5D^†^	0.79 (0.08) [0.6, 0.8, 0.9]	0.75 (0.1) [0.5, 0.77, 0.86]	0.1056

Values represent mean (SD) [min, median, max] for continuous variables and *n* (%) for categorical variables.

^†^Independent two-sample *t*-test, ^††^Wilcoxon's rank sum test, and ^*∗*^Chi-squared test.

BMI: body mass index; VAS: visual analogue scale; PGIC: patient global impression of change; PPT: pressure pain threshold; EQ-5D: Euro-Quality-of-Life Five Dimensions.

**Table 2:** Mean change in outcomes from baseline to each time interval.

	The laser acupuncture group			The sham laser acupuncture group			*P* value^††^
	(*n* = 28)			(*n* = 26)			
	Mean ± SD	95% CI^*∗*^	*P* value^†^	Mean ± SD	95% CI^*∗*^	*P* value^†^	
	(minimum, median, maximum)			(minimum, median, maximum)			
VAS							
Visit 1 (baseline)	44.64 ± 11.86 (30, 40, 75)			47.78 ± 10.95 (30, 40, 80)			
Visit 2	−2.68 ± 10.04 (−30, 0, 20)	[−5.91, 0.55]	0.0848	−5.56 ± 9.23 (−30, 0, 10)	[−8.59, −2.52]	<0.01	0.2743
Visit 3	−8.75 ± 10.42 (−30, −10, 10)	[−12.1, −5.4]	<0.001	−7.22 ± 12.58 (−40, −10, 20)	[−11.35, −3.09]	<0.01	0.6253
Visit 4 (f/u)	−11.07 ± 12.12 (−40, −10, 10)	[−14.97, −7.17]	<0.001	−12.78 ± 13.82 (−60, −10, 10)	[−17.31, −8.24]	<0.001	0.6281
PPT							
Visit 1 (baseline)	7.12 ± 2.27 (3, 7, 12)			6.56 ± 1.86 (4, 6, 12)			
Visit 2	−1.34 ± 1.66 (−5, −1, 1)	[−1.88, −0.81]	<0.001	−0.96 ± 1.26 (−4, −1, 2)	[−1.37, −0.55]	<0.001	0.3423
Visit 3	−1.3 ± 2.3 (−7, −1, 2)	[−2.04, −0.56]	<0.01	−0.76 ± 1.49 (−4, −1, 2)	[−1.24, −0.27]	<0.01	0.3060
Visit 4 (f/u)	−1.2 ± 2.21 (−6, −1, 3)	[−1.91, −0.49]	<0.01	−0.8 ± 1.55 (−4, −1, 2)	[−1.3, −0.29]	<0.01	0.4374
PGIC							
Visit 1 (baseline)	4 ± 0 (4, 4, 4)			4 ± 0 (4, 4, 4)			
Visit 4 (f/u)	−1 ± 0.72 (−2, −1, 0)	[−1.23, −0.77]	<0.001	−0.93 ± 0.78 (−3, −1, 1)	[−1.18, −0.67]	<0.001	0.7159
EQ-5D							
Visit 1 (baseline)	0.79 ± 0.08 (0.56, 0.81, 0.9)			0.75 ± 0.1 (0.51, 0.77, 0.86)			
Visit 4 (f/u)	0.03 ± 0.08 (−0.14, 0, 0.23)	[0.01, 0.06]	<0.05	0.04 ± 0.09 (−0.1, 0.04, 0.31)	[0.01, 0.07]	<0.01	0.5833

^†^Results of paired two-sample *t*-test for outcome variables within each group; ^††^results of independent two-sample *t*-test for outcome variables between groups.

^*∗*^Since all statistical analyses were set to the one-tailed test, 90% confidence intervals were provided.

VAS: visual analogue scale; PGIC: patient global impression of change; PPT: pressure pain threshold; EQ-5D: Euro-Quality-of-Life Five Dimensions; f/u: follow-up.
